# Analysis of COVID-19 Incidence and Protective Potential of Persisting IgG Class Antibodies against SARS-CoV-2 Infection in Hospital Staff in Poland

**DOI:** 10.3390/vaccines11071198

**Published:** 2023-07-04

**Authors:** Jadwiga Radziejewska, Jacek Arkowski, Robert Susło, Kamil Kędzierski, Magdalena Wawrzyńska

**Affiliations:** 1 Klodzko City Hospital, Ul. Szpitalna 1a, 57-300 Klodzko, Poland; 2 Centre for Preclinical Studies, Wrocław Medical University, Ul. Marcinkowskiego 1, 53-220 Wroclaw, Poland; 3 Epidemiology and Health Education Unit, Population Health Department, Wroclaw Medical University, Ul. Bujwida 44, 50-345 Wroclaw, Poland; 4 Department of Medical Emergencies, Wroclaw Medical University, Ul. Parkowa 34, 51-616 Wroclaw, Poland

**Keywords:** COVID-19 serological testing, SARS-CoV-2 PCR testing, COVID-19 immune response

## Abstract

The immune responses to both SARS-CoV-2 infection and vaccines are of key importance in prevention efforts. In April and May 2020, 703 study participants tested for COVID-19 by PCR tests were registered. In June and July 2020, they were examined for the presence of SARS-CoV-2 S1/S2 IgG. From October 2020 to January 2021, those among the study population with COVID-19 confirmed by PCR tests were registered, and the same group of participants was invited to be examined again for the presence of SARS-CoV-2 antibodies. In June 2020, antibodies were detected in only 88% of those who had PCR-confirmed COVID-19 in April–May 2020, which suggests that a significant proportion of persons in the Polish population do not produce antibodies after contact with SARS-CoV-2 antigens or rapidly lose them and reach levels below the lab detection limit. The levels of IgG class anti-SARS-CoV-2 antibodies were significantly lower among people who previously had COVID-19 than for those who had received COVID-19 vaccination, which confirms the high immunogenicity of the vaccines against COVID-19 in the Polish population. The study confirms that a detectable level of IgG class anti-SARS-CoV-2 antibodies cannot be considered a reliable marker of the presence and strength of COVID-19 immunity preventing individuals from acquiring SARS-CoV-2 infection.

## 1. Introduction

People in different medical occupations experience various types and durations of contact with patients and other staff members, which results in different risk levels of acquiring work-related COVID-19. Among medical professionals, nurses, most of whom are women, are the most exposed to infection [1]. According to psychological sources, women are generally more likely to comply with COVID-19 safety rules than men [2], as demonstrated by the former’s behavior in previous epidemics of infectious respiratory system diseases [3], such as influenza [4]. The perceived threat of infection is an important factor in decision-making [5], and compliance is higher for older people, who are generally intolerant of noncompliance with health-protective behaviors [6]. Younger individuals’ compliance with COVID-19 preventive measures is lower by comparison [7], and it should be kept in mind that medical personnel are not only involved in the general population, but they are part of it. Knowledge of and compliance with personal protective procedures are crucial for the safety of patients and personnel [8], and the declared levels of adherence to anti-COVID-19 rules are high among medical staff members [9,10], except when objective obstacles intervene, such as drastic supply shortages and extremely underequipped facilities [11].

Attempts to limit the spread of SARS-CoV-2 infection have been additionally complicated because a significant proportion of COVID-19 cases are mild or asymptomatic, especially for young and otherwise healthy individuals [12], which contrasts sharply with the grave illness and high mortality rates observed in elderly individuals with multiple chronic diseases [13].

Furthermore, a significant practical issue arises because many people do not have detectable levels of antibodies against SARS-CoV-2, either directly after or several months after contracting COVID-19. This translates into problems regarding the responses to and efficacy of COVID-19 vaccines and calls for evaluation of the scale of non-responders. It has been proven that, after the administration of inactivated SARS-CoV-2 vaccine, which closely resembles the natural antigens of the virus that originally circulated in the beginning of the pandemic, neutralizing antibodies are produced in 100% of previously infected people and only 91% of previously non-infected people. The antibody level drops significantly over the next several months, with a more pronounced drop among those who were previously not infected [14].

In practice, severe limitations hamper mass-scale attempts to evaluate the efficacy of immunization against COVID-19 gained from contracting the virus or being vaccinated against it [14]. This is because the available tests for evaluating humoral immunity to SARS-CoV-2—including the original plaque reduction neutralization test (PRNT) [15] and its simplified analogues, such as the cPass™ SARS-CoV-2 Neutralization Antibody Detection Kit by GenScript [16] and the Elecsys^®^ anti-SARS-CoV-2 S test by Roche Diagnostics [17]—are complex. As such, they could not be performed on a large enough sample of the Polish population. Consequently, measuring neutralizing antibody levels remains the most practically available test. It is comparatively easy to perform and allows us to detect the immune system response to viral infection and estimate the level of protection provided by a given vaccine against COVID-19 [18].

Current strategies for combating the lasting COVID-19 pandemic rely on the combined protective effect, with protection afforded by vaccine-induced immunity or natural immunization resulting from having been infected by COVID-19. The strategies based on vaccinations are highly cost efficient [19].

After several waves of the pandemic exposing the world’s population to SARS-CoV-2 and with a significant share of people already vaccinated against COVID-19, it is difficult to acquire current data characterizing the interaction of the previously unexposed population with the virus. These data would still be useful in studying the primary interactions of the virus with certain previously unexposed populations, including neonates and small children. Although the COVID-19 vaccines currently available in Poland are registered for administration in different age groups, including Comirnaty (Pfizer-BioNTech) [20], Spikevax (Moderna) [21], Vaxzevria (AstraZeneca) [22], Jcovden (Janssen-Cilag) [23], Nuvaxovid (Novavax) [24], and Valneva (Valneva) [25], initially small children could have not been vaccinated because there were not yet any registered vaccines for children below 5 years of age. Maternal antibodies only effectively protect newborns for approximately the first 6 months of life. This situation leaves small children practically without any innate protection against COVID-19, and multiple studies suggest that this results in many complications at different levels of severity. In response, starting on 19 October 2022, the European Medicines Agency recommended extending the use of Comirnaty and Spikevax, which target the original strain of SARS-CoV-2, by administering Comirnaty to children aged 6 months to 4 years and Spikevax to children aged 6 months to 5 years [26]. Putting this into medical practice required changing the registrations for both vaccines. Unfortunately, COVID-19 vaccine hesitancy is currently prevalent, even among parents of children 5 years old and above [27], as with other obligatory and elective vaccinations [28], although the vaccination of children against several diseases is required by law [29]. Despite the high hopes for the expected protection of infants derived from vaccinations in pregnancy [30] or the lactation period [31], any acceptable level of mother-acquired immunity against COVID-19 cannot be expected and the acceptance of vaccinations by pregnant women is low for other vaccines, including ones for influenza [32], even though they are well known to be safe and highly beneficial in pregnancy [33,34]. Nevertheless, the availability of COVID-19 vaccinations during pregnancy could potentially alleviate a significant amount of prenatal pandemic-related stress [35], supplementing the often ineffective, shielding-based protective strategies [36].

Consequently, the data concerning the spontaneous build-up and persistence of COVID-19 immunity among previously unexposed adults, including the data presented in this article, can be extrapolated to younger age groups to a large extent, adding to the base of practically useful knowledge on primary SARS-CoV-2 infection in neonates and young children. The data can also contribute to solving another public health and clinical practice-related problem, which is how long both kinds of immunity against COVID-19 remain and how the answer translates into easily measured levels of COVID-19 antibodies. This could be connected directly to the expected intensity of the waves of a future pandemic and the periods between the administrations of booster vaccine doses.

The objective of the study was to analyze the incidence of PCR-confirmed COVID-19 cases among personnel at a healthcare center in Poland in the initial phase of the pandemic. The age and sex distribution were evaluated, as well as the incidence and duration of immune response in the form of anti-SARS-CoV-2 IgG antibody levels following active disease or vaccination and the influence of antibody levels on the risk of contracting COVID-19 disease.

## 2. Materials and Methods

The study was approved by the Institutional Ethics Committee of Wrocław Medical University in Wrocław, Poland (protocol code: KB-1014/2021; date of approval: 13 December 2021) and was conducted by retrospective statistical analysis of anonymized data collected between June 2020 and February 2021. The data were generated from hospital-coordinated pandemic-limiting actions that involved repetitive cross-sectional testing for SARS-CoV-2 by using PCR and SARS-CoV-2 IgG antibodies for 703 staff members of Kłodzko County Hospital in Poland. The staff members consented to the use of their anonymized test data for scientific analysis and publication. The data analyzed for this study were collected in four phases of cross-sectional testing aimed at detecting either active SARS-CoV-2 infection or post-COVID-19 immunity. In the first phase, during the first wave of the pandemic in April–May 2020, COVID-19 cases confirmed by PCR tests among the study population were registered. In the second phase, from June to July 2020, 703 participants were examined for the presence of SARS-CoV-2 S1/S2 IgG antibodies using the LIAISON^®^ SARS-CoV-2 S1/S2 IgG test by Diasorin (Saluggia, Italy). In the third phase, from October 2020 to January 2021, the second wave of the pandemic, COVID-19 cases confirmed by PCR in the study population were registered. In the fourth and final phase of the study, in February 2021, the same group of participants was invited to be examined for the presence of SARS-CoV-2 S1/S2 IgG antibodies using the same LIAISON^®^ SARS-CoV-2 S1/S2 IgG test.

## 3. Results

The study group (703 participants) was composed of 559 (79.55%) women and 144 (20.45%) men, a proportion that was unavoidable, as more women than men are employed at the hospital.

Among the study group, 289 (41.11%) of the participants were 50–59 years old, 154 (21.91%) were 40–49 years old, and 153 (21.76%) were 60–69 years old, with lower percentages of other age groups: 66 (9.39%) were 30–39 years old, 23 (3.27%) were 20–29 years old, 11 (1.56%) were 70–79 years old, and 7 (1.00%) were 80 years old or older.

However, only 10.5% of the initial group of participants consented to continue taking part in this fourth phase of the study: 10.73% women and 9.72% men; 19.48% in the age group 40–49 years, 11.42% in the age group 50–59 years, 7.58% in the age group 30–39 years, 4.35% in the age group 20–29 years, 3.27% in the age group 60–69 years, and none in the oldest age group. This did not significantly change the proportions of women (81.08% vs. initial 79.55%) and men (18.92% vs. initial 20.45%), but it concentrated the study group into the age groups 50–59 years (44.59% vs. initial 41.11%) and 40–49 years (40.54% vs. initial 21.91%), as indicated in Table 1 and Table 2.

In the study population, among the individuals who contracted COVID-19, no significant differences were found between sex groups or age groups, as can be seen in Table 3 and Table 4.

Of the 703 participants, 141 had a positive PCR (PCR+) test, and 562 had a negative PCR (PCR−) test. The median age of the PCR+ group was 51 (range: 28–72) compared to 54 (range 22–91) in the PCR− group.

The observed COVID-19 incidence was highest in the age groups 30–40 years and 40–50 years, at 27.3 and 23.4%, respectively, as demonstrated in Table 5.

COVID-19 was confirmed in only 4% of the individuals in the study group in April–May 2020. In June 2020, IgG class anti-SARS-CoV-2 antibodies were detected in over 7.2% of subjects in this group, as shown in Table 6.

In our study, IgG class anti-SARS-CoV-2 antibodies were detected in only 88% of the individuals in June 2020 who contracted PCR-confirmed COVID-19 in April–May 2020, as demonstrated in Table 7.

In the study group, IgG class anti-SARS-CoV-2 antibody levels were significantly lower among people who had previously had COVID-19 (mean: 53.60) than those who received a COVID-19 vaccination (mean: 245.72), as seen in Table 8.

The results show that contracting SARS-CoV-2 infection in April–May 2020 was associated with a reduced risk of contracting the disease in October 2020 to January 2021, as seen in Table 9. On the other hand, in June 2020, the level of IgG class anti-SARS-CoV-2 antibodies detected in people who did not contract PCR-confirmed COVID-19 in the preceding months and were asymptomatic (antibodies without confirmed disease) were not associated with contracting the disease in October 2020 to January 2021, as seen in Table 10.

## 4. Discussion

The results revealed no significant differences in the proportions of individuals who contracted COVID-19 based on sex or age. This is consistent with the findings of other studies on COVID-19 incidence among healthcare workers [37] but different from the findings of the studies concerning the general population, where males were reported to be more affected than females [38]. The lack of difference for healthcare workers can be attributed to their commonly high level of professional medical training that was indifferent to sex or age.

In this study, COVID-19 incidence was highest in two age groups, 30–40 years and 40–50 years, at 27.3 and 23.4%, respectively. The study results are consistent with the literature concerning healthcare workers [39,40,41] but different from published data on the general population [41], which may result from healthcare workers’ intensive professional activity and the need to combine more social roles during the pandemic than the general population. As the study involved a group of healthcare workers that differs in sex and age structure from the general population, its results are not directly generalizable.

Greek studies of the prevalence of COVID-19 indicated that in the general population during the first wave of the COVID-19 pandemic period, the incidence was low, resulting in 0.45% people with IgG class anti-SARS-CoV-2 antibodies [B]. The respective seroprevalence in the Polish general population was twice as high—0.93% [42]. In contrast, in our study population of healthcare workers, COVID-19 was confirmed in 4% of individuals in April–May 2020, and in June 2020, IgG class anti-SARS-CoV-2 antibodies were detected in over 7.5% of subjects. This seems to confirm the literature data, pointing to significant proportions of low symptomatic or asymptomatic cases or cases with atypical symptoms among people with COVID-19 who spread the infection easily and unnoticed [43]. The large proportion of asymptomatic cases significantly biased both the perception of the real extent of SARS-CoV-2 circulation in the Polish population and estimations of the infection/fatality ratio [44].

While SARS-CoV-2 polymerase chain reaction with reverse transcription (RT-PCR) detection is a tool of choice in the diagnosis of acute COVID-19 infection, serological assays serve as the primary means for confirming the humoral immune response to SARS-CoV-2 resulting from contact with its antigens in the loosely defined past. It was confirmed that IgG class anti-SARS-CoV-2 antibodies directed against spike (S) protein and its receptor-binding domain (RBD) were present in about 90.9% of people who succumbed to COVID-19 up to 6 months after the onset of symptoms, while their ability to neutralize live SARS-CoV-2 virus needs to be individually assessed for even limited future immune response estimation [45]. In our study in June 2020, IgG class anti-SARS-CoV-2 antibodies were detected in only 88% of individuals who had contracted COVID-19 as confirmed by PCR tests in April–May of that year. This finding confirms the importance of the problem of the people, often called non-responders [46,47], who do not produce antibodies after contact with SARS-CoV-2 antigens. Alternatively, this may be explained by a rapid decrease in initially achieved antibody levels to values below lab detection limits in a significant percentage of the population [43]. The ability of the immune system to protect against a severe course of the disease and the response of these people to standard COVID-19 vaccinations remain to be determined.

In numerous studies, a broad variety in antibody response levels to infection with SARS-CoV-2 was reported, while the completion of the anti-COVID-19 vaccine series typically leads to consistent and more intense initial antibody response but with a faster decline in antibody levels [48]. It was reported that vaccines manufactured both by Pfizer and Moderna tend to generate similar peak levels of anti-SARS-CoV-2 antibodies, but with the Moderna vaccine, individuals’ levels rise more quickly and decline more slowly than with the Pfizer vaccine. Both the Moderna vaccine recipients after half a year and patients who were hospitalized with severe COVID-19 six months before testing tend to have antibody levels higher than those of Pfizer vaccine recipients after six months [49]. In our study group, IgG class anti-SARS-CoV-2 antibody levels were significantly lower among people who had previously had COVID-19 than among those who received COVID-19 vaccination, which is consistent with the reports of other researchers [50]. This confirms the high immunogenicity of vaccines against COVID-19 reported in the Polish population in other studies [51] for the immune response evoked by the vaccine and the magnitude of the response sustained over time [52].

The study results show that contracting SARS-CoV-2 infection in April–May 2020 was associated with a significantly reduced risk of contracting the disease in October 2020 to January 2021. Nevertheless, the level of IgG class anti-SARS-CoV-2 antibodies detected in June 2020 among people who did not contract PCR-confirmed COVID-19 in the preceding months and were asymptomatic (antibodies without confirmed disease) was not correlated with contracting the disease in October 2020 to January 2021. This confirms the previous observation in other studies that immunization by either active COVID-19 infection or COVID-19 vaccination results in immunity that lasts a few months and provides protection against a severe clinical course of the disease but gives only limited protection from infection [47]. The study results also confirm the results of other studies [49] that a detectable level of IgG class anti-SARS-CoV-2 antibodies cannot be treated as a reliable marker of the presence and strength of COVID-19 immunity preventing the individual from acquiring a SARS-CoV-2 infection.

## 5. Conclusions

COVID-19 incidence among hospital workers was highest in two age groups, 30–40 years and 40–50 years, at 27.3 and 23.4%, respectively, which may stem from intensive professional activity and an accompanying involvement in other social roles during the pandemic, unlike the general population.

In the study population, COVID-19 was confirmed for only 4% of the individuals in April–May 2020, while in June 2020, IgG class anti-SARS-CoV-2 antibodies were detected for over 7.5% of subjects in this group, data that confirm the significant proportions of low-symptomatic or asymptomatic cases or cases with atypical symptoms among the people ill with COVID-19.

In our study, in June 2020, IgG class anti-SARS-CoV-2 antibodies were detected in only 88% of individuals who contracted PCR-confirmed COVID-19 in April–May, which suggests that a significant proportion of people in the Polish population do not produce antibodies after contact with SARS-CoV-2 antigens or rapidly lose them, reaching levels below the lab detection limit.

In the study group, IgG class anti-SARS-CoV-2 antibody levels were significantly lower among people who had previously had COVID-19 than among those who received COVID-19 vaccination, which confirms the high immunogenicity of vaccines against COVID-19 in the Polish population.

The study shows that contracting SARS-CoV-2 infection in April–May 2020 was associated with a significantly reduced risk of contracting the disease in October 2020 to January 2021, but the level of IgG class anti-SARS-CoV-2 antibodies detected in June 2020 among people who did not contract PCR-confirmed COVID-19 in the preceding months (antibodies without confirmed disease) was not correlated with contracting the disease a few months later in October 2020 to January 2021. This supports the thesis that immunization by either active COVID-19 infection or COVID-19 vaccination results in immunity that approximately lasts only a few months and protects against a severe clinical course of the disease but offers only limited protection from infection. The study results also confirm that a detectable level of IgG class anti-SARS-CoV-2 antibodies cannot be treated as a reliable marker of the presence and strength of COVID-19 immunity to the SARS-CoV-2 infection.

## Figures and Tables

**Table 1:** Sex division of study group during phases 1–4.

Sex	Phase 1 *n* (%)	Phase 2 *n* (%)	Phase 3 *n* (%)	Phase 4 *n* (%)
F	559 (79.55)	559 (79.55)	559 (79.55)	60 (81.08)
M	144 (20.45)	144 (20.45)	144 (20.45)	14 (18.92)
Total	703 (100.0)	703 (100.0)	703 (100.0)	74 (100.0)

**Table 2:** Age division of study group during phases 1–4.

Age (Years)	Phase 1 *n* (%)	Phase 2 *n* (%)	Phase 3 *n* (%)	Phase 4 *n* (%)
20–29	23 (3.27)	23 (3.27)	23 (3.27)	1 (1.35)
30–39	66 (9.39)	66 (9.39)	66 (9.39)	5 (6.76)
40–49	154 (21.91)	154 (21.91)	154 (21.91)	30 (40.54)
50–59	289 (41.11)	289 (41.11)	289 (41.11)	33 (44.59)
60–69	153 (21.76)	153 (21.76)	153 (21.76)	5 (6.76)
70–79	11 (1.56)	11 (1.56)	11 (1.56)	0 (0.0)
80 or more	7 (1.00)	7 (1.00)	7 (1.00)	0 (0.0)
Total	703 (100.0)	703 (100.0)	703 (100.0)	74 (100.0)

**Table 3:** Sex distribution among study participants with positive and negative SARS-CoV-2 PCR test results.

Sex	PCR+ *n* (%)	PCR− *n* (%)	Test
F	116 (82.3)	443 (78.8)	Fisher’s
Total	141 (100.0)	562 (100.0)	

**Table 4:** Age distribution among study participants with positive and negative SARS-CoV-2 PCR test results.

Age	PCR+ *n* (%)	PCR− *n* (%)	Test
20–29	2 (1.4)	21(3.7)	Fisher’s
30–39	18 (12.8)	48 (8.5)	*p* = 0.091
40–49	36 (25.5)	118 (21.0)	
50–59	62 (44.0)	227 (40.4)	
60–69	21 (14.9)	132 (23.5)	
70–79	2 (1.4)	9 (1.6)	
80 or more	0 (0.0)	7 (1.2)	
Total	141 (100.0)	562 (100.0)	

**Table 5:** Incidence of COVID-19 in different age groups.

Age	PCR+ *n* (%)	PCR− *n* (%)	Participants in Age Group *n* (%)
20–29	2 (8.7)	21 (91.3)	23 (100.0)
30–39	18 (27.3)	48 (72.7)	66 (100.0)
40–49	36 (23.4)	118 (76.6)	154 (100.0)
50–59	62 (21.5)	227 (78.5)	289 (100.0)
60–69	21 (13.7)	132 (86.3)	153 (100.0)
70–79	2 (18.2)	9 (81.8)	11 (100.0)
80 or more	0 (0.0)	7 (100.0)	7 (100.0)

**Table 6:** Results of COVID-19 tests in different stages of the study.

Study Phase	Positive *n* (%)	Negative *n* (%)	Total Participants *n* (%)
SARS-CoV-2 PCR result April–May 2020 or October 2020 to January 2021.	141 (20.0)	562 (80.0)	703 (100.0)
SARS-CoV-2 PCR result April–May 2020.	28 (4.0)	675 (96.0)	703 (100.0)
SARS-CoV-2 PCR result October 2020 to January 2021.	113 (16.1)	590 (83.9)	703 (100.0)
SARS-CoV-2 IgG result June 2020.	51 (7.25)	652 (92.5)	703 (100.0)
SARS-CoV-2 IgG result February 2021.	69 (93.2)	5 (6.8)	74 (100.0)

**Table 7:** Comparison between contracting COVID-19 (confirmed by PCR) in April–May 2020 and antibody levels (IgG) in June 2020.

	April–May 2020 SARS-CoV-2 PCR Positive *n* (%)	April–May 2020 SARS-CoV-2 PCR Negative *n* (%)	Test
June 2020 SARS-CoV-2 IgG positive	23 (88.5)	28 (4.3)	Fisher’s *p* < 0.001
June 2020 SARS-CoV-2 IgG negative	3 (11.5)	629 (95.7)	
TOTAL	26 (100.0)	657 (100.0)	

**Table 8:** Comparison between IgG class SARS-CoV-2 antibody levels following COVID-19 and vaccination (W = 124.5, *p* < 0.001).

Variable	*n*	Mean	SD	Median	Min	Max
IgG class anti-SARS-Co-2 antibody levels about 2 months after confirmed COVID-19 disease in April–May 2020	11	53.6	53.7	24.3	3.8	158.0
IgG class anti-SARS-CoV-2 antibody levels after COVID-19 vaccination in February 2021	68	245.7	162.9	315.5	8.3	401.0

**Table 9:** Comparison of PCR-confirmed SARS-CoV-2 in April–May 2020 vs. October 2020 to January 2021.

	October 2020 to January 2021 SARS-CoV-2 PCR Positive *n* (%)	October 2020 to January 2021 SARS-CoV-2 PCR Negative *n* (%)	Test
April–May 2020 SARS-CoV-2 PCR positive	0 (0.0)	28 (100.0)	Fisher’s *p* = 0.015
April–May 2020 SARS-CoV-2 PCR negative	113 (16.7)	563 (83.3)	

**Table 10:** Comparison between elevated IgG levels in June 2020 in people with asymptomatic non-confirmed COVID-19 in April–May 2020 and contracting the disease in October 2020 to January 2021.

	October 2020 to January 2021 SARS-CoV-2 PCR Positive *n* (%)	October 2020 to January 2021 SARS-CoV-2 PCR Negative *n* (%)	Test
June 2020 SARS-CoV-2 IgG positive	4 (14.3)	24 (85.7)	Fisher’s *p* = 1
June 2020 SARS-CoV-2 IgG negative	103 (16.4)	526 (83.6)	

## Data Availability

The research data are available from the authors on request.
